# Fusion Protein Cleavage Site Containing Three Basic Amino Acids Attenuates Newcastle Disease Virus in Chicken Embryos: Use as an *in ovo* Vaccine

**DOI:** 10.3389/fmicb.2022.812289

**Published:** 2022-03-21

**Authors:** Helong Feng, Yu Shang, Li Li, Xiuxiu Sun, Sanling Fan, Xiangfei Ren, Yingying Xu, Zhe Zeng, Xingxing Hu, Guofu Cheng, Guoyuan Wen

**Affiliations:** ^1^Division of Veterinary Pathology, College of Veterinary Medicine, Huazhong Agricultural University, Wuhan, China; ^2^Institute of Animal Husbandry and Veterinary Sciences, Hubei Academy of Agricultural Sciences, Wuhan, China; ^3^Hubei Provincial Key Laboratory of Animal Pathogenic Microbiology, Wuhan, China; ^4^Key Laboratory of Prevention and Control Agents for Animal Bacteriosis (Ministry of Agriculture), Wuhan, China

**Keywords:** Newcastle disease virus, *in ovo* vaccine, fusion protein, cleavage site, attenuation

## Abstract

*In ovo* vaccination is an attractive immunization strategy for the poultry industry. However, although most live Newcastle disease virus (NDV) vaccine strains, such as LaSota and V4, can be used after hatching, they are pathogenic to chicken embryos when administered *in ovo*. We have previously reported that NDV strain TS09-C is a safe *in ovo* vaccine in specific-pathogen-free and commercial chicken embryos because it is attenuated in chicken embryos. However, the molecular basis of its attenuation is poorly understood. In this study, we firstly evaluated the safety of chimeric NDV strains after exchanging genes between strains TS09-C and LaSota as *in ovo* vaccines, and demonstrated that the attenuation of NDV in chicken embryos was dependent upon the origin of the fusion (F) protein. Next, by comparing the F protein sequences of TS09-C strain with those of LaSota and V4 strain, the R115 in cleavage site and F379 were found to be unique to TS09-C strain. The mutant viruses were generated by substituting one or two amino acids at position 115 and 379 in the F protein, and their safety as *in ovo* vaccine was evaluated. Mutation in residue 379 did not affect the viral embryonic pathogenicity. While the mutant virus rTS-2B (R115G mutation based on the backbone of TS09-C strain) with two basic amino acids in F cleavage site, was pathogenic to chicken embryos and similar with rLaSota in its tissue tropism, differing markedly from rTS09-C with three basic amino acids in F cleavage site. Together, these findings indicate that the F protein cleavage site containing three basic amino acids is the crucial determinant of the attenuation of TS09-C in chicken embryos. This study extends our understanding of the pathogenicity of NDV in chicken embryos and should expedite the development of *in ovo* vaccines against NDV.

## Introduction

Newcastle disease (ND) is one of the most important infectious diseases of birds, and has a substantial economic impact on the poultry industry (Zhu et al., 2016; Chi et al., 2019). Newcastle disease virus (NDV), the causative agent of ND, is classified into lentogenic, mesogenic, and velogenic strains based on its pathogenicity in chickens (Alexander and Allan, 1974). Its genome is a negative-sense, single-stranded RNA molecule that encodes six structural proteins: nucleocapsid protein (NP), phosphoprotein (P), matrix protein (M), fusion protein (F), hemagglutinin-neuraminidase (HN), and large polymerase protein (L) (Dimitrov et al., 2019).

Immunization against NDV with live vaccines is a common strategy in many countries. The conventional methods of vaccination, including spraying, in drinking water, and in feed, can result in inconsistent vaccine delivery and consequent poor vaccine efficacy (Mebatsion et al., 2001; Sokale et al., 2017; Wen et al., 2017). Where possible, these methods are being replaced by *in ovo* injection, which is a faster, more effective, and uniform method of vaccine delivery (Negash et al., 2004; Williams and Zedek, 2010). Sharma and Burmester (1982) first reported that *in ovo* vaccination could be used effectively to protect birds against Marek’s disease (MD). In 1995, the automated Inovoject^®^ system was used to immunize 55% of broiler embryos against MD in North America (Johnston et al., 1997). At present, vaccines for several poultry diseases have been approved for *in ovo* immunization, including those for MD, infectious bursal disease, fowl pox, ND, and coccidiosis (Peebles, 2018).

Although NDV vaccines had been approved for *in ovo* delivery, they were not yet commercially available (Peebles, 2018). The NDV B1 strain could kill embryos when it was used to vaccinate 18-day-old chicken embryos (Ahmad and Sharma, 1992). When vaccinated with the NDV LaSota strain, specific-pathogen-free (SPF) chicken embryos showed only 24% hatchability, although the optimized LaSota mutant strain had an improved survival rate, it still displayed residual pathogenicity in SPF chicken embryos (Mast et al., 2006). A 30 SPF chicken embryos were vaccinated with NDV Clone-30 strain, only one chicken survived during the 21-day observation period (Ramp et al., 2012). The NDV V4 strain was also unsafe as an *in ovo* vaccine, and poor survival and severe histopathological lesions in hatched SPF and commercial chickens had been observed (Wen et al., 2017; Fan et al., 2020). Although most NDV vaccine strains were safely administered to hatched chickens, they could not be used for *in ovo* vaccination because of their embryonic lethality (Mast et al., 2006).

We previously developed a new NDV strain, TS09-C, by serial passaging strain V4 in BHK-21 cells. TS09-C was an avirulent strain, with an intracerebral pathogenicity index (ICPI) of 0.00 and a mean death time (MDT) > 168 h (Wen et al., 2013, 2016). The TS09-C strain was safe and immunogenic when used as an *in ovo* vaccine for SPF and commercial chicken embryos, in which both the hatchability and survival rates were higher than 90% after vaccination (Wen et al., 2017; Fan et al., 2020). However, it is unclear why strain TS09-C is attenuated when used as an *in ovo* vaccine in chicken embryos.

To explore the molecular basis of the attenuation of TS09-C in chicken embryos, chimeric viruses (exchanging genes between NDV strains TS09-C and LaSota) were used, mutant viruses were constructed with site-directed mutagenesis, and their safety as *in ovo* vaccines was evaluated. Our results showed that an F protein cleavage site (Fcs) containing three basic amino acids was the crucial determinant of the attenuation of TS09-C in chicken embryos.

## Materials and Methods

### Animals and Ethics Statement

Specific-pathogen-free Leghorn chicken embryos were purchased from Merial-Vital, Beijing, China. The embryos were hatched in a contained environment at 37.5°C and ∼60% humidity, and raised in negative-pressure isolators. All the animal experiments were approved (permit number: 30/2020) and supervised by the Institutional Animal Care and Use Committee of the Hubei Academy of Agriculture Sciences, Wuhan, China.

### Cells and Viruses

BHK-21 cells were maintained in Dulbecco’s modified Eagle’s medium (DMEM) (Gibco, United States) containing 10% fetal bovine serum (FBS) (Gibco) and incubated at 37°C under 5% CO_2_. NDV strains TS09-C and LaSota were obtained from the Pathogen Repository Bank at Hubei Academy of Agriculture Sciences. The GenBank accession numbers of NDV strains LaSota, V4, and TS09-C are JF950510, JX524203.1, and JX110635, respectively. Ten chimeric NDV strains, rLS-T-A, rLS-T-B, rLS-T-C, rTS-L-A, rTS-L-B, rTS-L-C, rTS-L-HN, rTS-L-F, rLS-T-HN, and rLS-T-F, were constructed previously (Wen et al., 2017).

### Construction of Mutant Infectious cDNA Clones

The infectious cDNA clones (ICs) of NDV strain TS09-C and LaSota, named pTS and pLS, respectively, were constructed previously (Hu et al., 2011; Wen et al., 2015). pTS or pLS were used as the backbone upon which to construct a series of mutant ICs at positions 115 and 379 in F gene. Briefly, the vector fragment was amplified by using the backbone ICs as a template and vector-specific primers to exclude the gene F fragment (4,890–6,306 nt) or (5,655–6,306 nt). The mutated F gene (4,875–6,321 nt) or (5,639–6,321 nt) were amplified by multiple over-lapping polymerase chain reaction (PCR), using the backbone ICs as a template and specific primers containing the corresponding mutations. Subsequently, the mutant ICs were generated by ligation of the two PCR products, mutated F gene and vector fragment, using In-Fusion^®^ Snap Assembly (Takara, United States). The mutated full-length ICs containing R115G, F379L, R115G & F379L, G115R, L379F, and G115R & L379F substitutions were designated pTS-2B, pTS-a, pTS-2B-a, pLS-3B, pLS-b, and pLS-3B-b, respectively (Figure 2). The entire genomes of the infectious cDNA clones were sequenced to verify that the rest of the genes remained unchanged after mutagenesis.

### Rescue of Newcastle Disease Virus Mutants

The NDV mutants were rescued by co-transfecting MAV-T7-infected BHK-21 cells with each infectious cDNA clone and an NP-, P-, or L-expressing plasmid, using Lipofectamine 3000 (Invitrogen, United States), as described previously (Wen et al., 2015). The rescued viruses were amplified by inoculating 100 μL of the cell lysate into the allantoic cavities of 9-day-old SPF chicken embryos and incubating the embryos at 37.5°C and ∼60% humidity. At 4 days post-inoculation (dpi), the allantoic fluids were harvested and the rescued viruses were examined with a hemagglutination (HA) assay using 1% chicken red blood cells. The mutant viruses were passaged three times in chicken embryos to test their stability.

### Virus Titration and Pathogenicity Tests

The titers of the chimeric NDV strains were examined by HA assay, 50% tissue culture infectious dose (TCID_50_) assay on BHK-21 cells in the presence of 0.2 μg/mL TPCK–trypsin, and median embryo infectious dose (EID_50_) assay in 10-day-old embryonated SPF chicken eggs. The pathogenicity of the chimeric NDV strains was determined as MDT in 10-day-old embryonated SPF chicken embryos and with ICPI in 1-day-old SPF chickens (Alexander and Allan, 1974).

### *In ovo* Vaccine

The method of *in ovo* vaccination has been described previously (Wen et al., 2017). Briefly, 18-day-old chicken embryos were cleaned with 75% ethanol, and inoculated with one NDV strain at a dose of 10^3^.^0^ EID_50_/egg or an equal volume of phosphate-buffered saline (PBS) via the amniotic route with a 38 mm 23G needle at a depth of 1 inch. The vaccinated eggs were sealed with adhesive and hatched at separate incubators. The proportions of vaccinated eggs that hatched successfully without assistance and survived to 14 days post-hatching (dph) were recorded for each group. Since lentogenic pathotypes such as B1 and LaSota administered to 1- to 7-day-old chicks, can cause reduced food intake and respiratory distress, or sneezing (Terregino and Capua, 2009), the clinical signs of in-ovo vaccinated birds were scored. Over a period of 14 days after hatching, the vaccinated birds were scored clinically as: normal = 0, slightly sick (reduced food intake or respiratory distress) = 1, severely sick (reduced food intake and respiratory distress) = 2, dead = 3. Blood samples were collected from five birds in each group to determine the NDV hemagglutinin-inhibition (HI) titers on the day of 1, 7, 14, and 21 dph. The antigen used for HI detection was the LaSota strain. At 2 and 4 dpi, three birds from each group were sacrificed randomly. The lung, trachea, duodenum, liver, kidneys, muscles, and spleen were collected from all the killed birds, and viral titer were determined by TCID_50_ assay. Parts of the lung and tracheal tissues collected at 4 dpi were fixed in 4% paraformaldehyde, paraffin embedded, sectioned and stained with hematoxylin–eosin, and analyzed microscopically.

### Statistical Analysis

All data were analyzed with an independent-samples *t* test. Statistical significance was defined as: not significant (ns), *p* > 0.05; significant, *p < 0.05; very significant, ^**^p < 0.01.

## Results

### Attenuation Determinant of TS09-C in Chicken Embryos Is Located Within the Region Spanning the Fusion and Hemagglutinin-Neuraminidase Genes

To identify the attenuation determinant of TS09-C in chicken embryos, six chimeras constructed with fragment replacements between strains TS09-C and LaSota were used: rLS-T-A, rLS-T-B, rLS-T-C, rTS-L-A, rTS-L-B, and rTS-L-C (fragment A contains NP, P, and M genes; B contains F and HN genes; C contains L gene) [as generated in Wen et al. (2016)]. Eighteen-day-old SPF chicken embryos were inoculated with the six chimeric viruses as *in ovo* vaccines, and their safety was evaluated. As shown in Table 1, among the three chimeric viruses based on the LaSota background, only rLS-T-B (the survival rate was 86.7%) showed attenuation in 18-day-old SPF chicken embryos. Among the three chimeric viruses based on the TS09-C background, only rTS-L-B (the survival rate was 20%) was pathogenic in chicken embryos. Genomic fragment B contains both the F and HN genes. Therefore, the virulence determinant of NDV strain TS09-C in chicken embryos is located within the region spanning the F and HN genes.

**TABLE 1:** Hatchability and survival rates of SPF chickens vaccinated *in ovo* with different NDV chimeras.

Vaccines	Dosage (lg EID_50_)	Eggs	%hatched	%survival (14 day post-hatch)
PBS	–	30	93.3(28/30)	93.3(28/30)
rLaSota	3.0	30	36.7(11/30)	0(0/30)
rLS-T-A	3.0	30	40(12/30)	13.3(4/30)
rLS-T-B	3.0	30	90(27/30)	86.7(26/30)
rLS-T-C	3.0	30	36.7(11/30)	16.7(5/30)
rTS09-C	3.0	30	93.3(28/30)	90(27/30)
rTS-L-A	3.0	30	93.3(28/30)	86.7(26/30)
rTS-L-B	3.0	30	43.3(13/30)	20(6/30)
rTS-L-C	3.0	30	90(27/30)	86.7(26/30)
rLS-T-F	3.0	30	93.3(28/30)	93.3(28/30)
rLS-T-HN	3.0	30	43.3(13/30)	16.7(5/30)
rTS-L-F	3.0	30	46.7(14/30)	23.3(7/30)
rTS-L-HN	3.0	30	90(27/30)	86.7(26/30)

*Fragment A contains the NP, P, and M genes; fragment B contains the F and HN genes; fragment C contains the L gene. Eighteen-day-old SPF chicken embryos were inoculated with the same dose of different NDV chimeras.*

### Fusion Protein Is the Attenuation Determinant of TS09-C in Chicken Embryos

To narrow the region in which the virulence determinant occurs, 18-day-old SPF chicken embryos were inoculated with different NDV chimeras: rTS-L-HN, rTS-L-F, rLS-T-HN, or rLS-T-F [as generated in Wen et al. (2016)]. As shown in Table 1, the survival rates of the NDV chimeras rTS-L-F (F gene from the LaSota strain) and rTS-L-HN (HN gene from strain LaSota) were 23.3 and 86.7%, respectively, at 14 dph. The survival rates of NDV chimera rLS-T-F (F gene from the TS09-C strain) and rLS-T-HN (HN gene from strain TS09-C) were 93.3 and 16.7%, respectively, at 14 dph. Therefore, NDV chimeras containing the F gene of TS09-C were safe for SPF chicken embryos. These results demonstrate that the F protein is the attenuation determinant of TS09-C in chicken embryos.

### Fcs Is the Crucial Attenuation Determinant of TS09-C in Chicken Embryos

**FIGURE 1:**
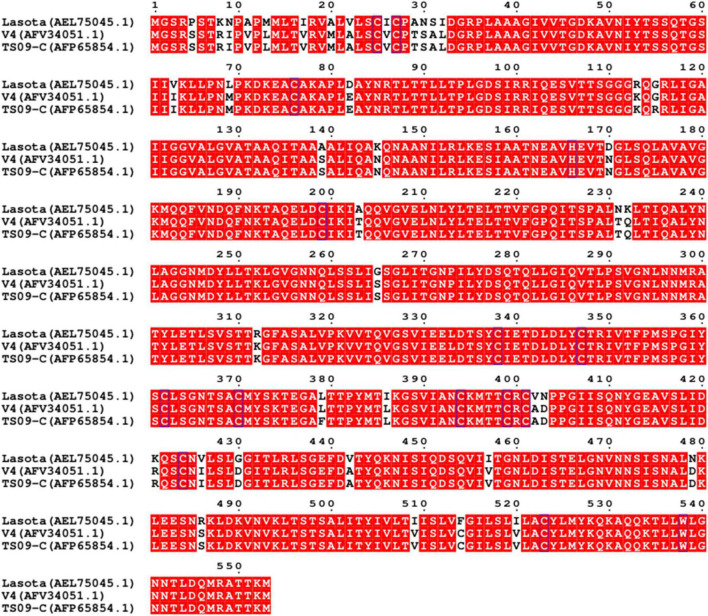
Amino acid sequence alignment of the F proteins of NDV strains LaSota, V4, and TS09-C. The amino acid sequence alignment of F proteins from NDV strains LaSota, V4, and TS09-C was constructed at the website ESPript 3.0 (https://espript.ibcp.fr/ESPript/cgi-bin/ESPript.cgi). The sites without red highlighting contain different amino acids.

To confirm the key site in F protein that causes the attenuation of TS09-C in chicken embryos, the F protein sequences of two NDV strains LaSota and V4 with embryonic lethality were compared with that of TS09-C strain with embryonic attenuation. As shown in Figure 1, 39 amino acid sites differed between the F proteins of NDV strains LaSota, V4, and TS09-C. Of these, 37 amino acids were same in strains V4 and TS09-C, the other two amino acids located at 115 and 379 were unique to strain TS09-C.

**FIGURE 2:**
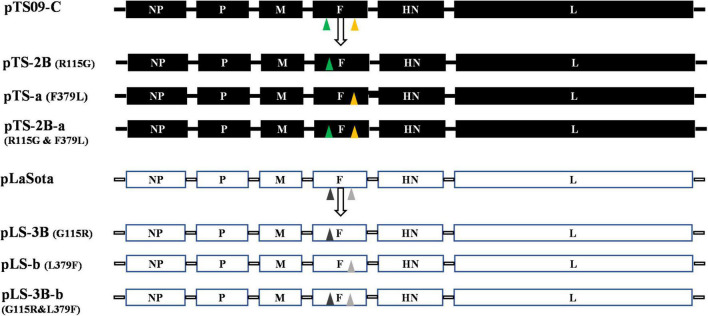
Schematic representation showing the construction of mutant NDVs. The mutated ICs containing the R115G and/or F379L substitution(s) were designated as pTS-2B, pTS-a, and pTS-2B-a, respectively, according to the mutation sites. The mutated ICs containing the G115R and/or L379F substitution(s) were designated as pLS-3B, pLS-b, and pLS-3B-b, respectively. The triangles with different colors indicate the different mutation.

To confirm the effect of R115G and F379L on the attenuation of TS09-C in chicken embryo, six mutate ICs of NDV containing different substitutions based on the ICs of strain TS09-C and LaSota were designated, and named as pTS-a (F379L), pTS-2B (R115G), pTS-2B-a (F379L and R115G), pLS-b (L379F), pLS-3B (G115R) and pLS-3B-b (L379F and G115R) (Figure 2). Finally, mutants rTS-a, rTS-2B, rTS-2B-a, and rLS-b were successfully rescued, but rLS-3B and rLS-3B-b were not. The biological characteristics of the successfully rescued mutant viruses were evaluated. As shown in Table 2, mutant virus rLS-b showed similar pathogenicity to that of strain rLaSota (ICPI = 0.34 and MDT = 108 h), and rTS-a was similar to its parental strain rTS09-C (ICPI = 0.00 and MDT > 168 h). The pathogenicity of chimeric virus rTS-2B (ICPI = 0.15 and MDT = 112 h) was similar to that of strain LaSota, but not to that of TS09-C. The amino acid mutation R115G was located at the Fcs. These results imply that Fcs is the crucial attenuation determinant of TS09-C in chicken embryos.

**TABLE 2:** Pathogenicity and growth titer of different NDV mutants.

Virus	MDT(h)	ICPI	HA titer (log_2_)	Virus titer	
				Allantoic fluid (lg EID_50_/mL)	BHK21 (lg TCID_50_/mL)
rTS09-C	>168	0.00	8.67 ± 1.15	9.50 ± 0.50	9.03 ± 0.34
rTS-2B	112	0.15	8.33 ± 0.36	9.0 ± 0.20	9.0 ± 0.20
rTS-a	>168	0.00	8.48 ± 0.78	9.25 ± 0.20	9.17 ± 0.31
rTS-2B-a	118	0.10	9.17 ± 1.08	9.08 ± 0.42	9.08 ± 0.24
rLaSota	108	0.34	11.33 ± 0.58	9.25 ± 0.58	8.21 ± 0.68
rLS-b	108	0.20	10.8 ± 0.69	9.17 ± 0.31	8.5 ± 0.20

*Data shown represent the averages of three independent experiments (means ± SD, n = 3).*

**FIGURE 3:**
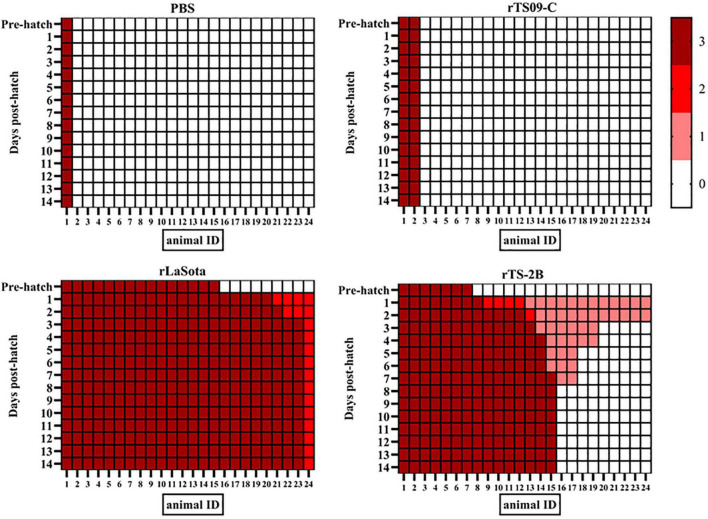
Overview of the clinical features of chickens *in ovo* vaccinated with NDV. The clinical scores for 24 birds in each group. At each observation point, each bird was scored: normal = 0, slightly sick (reduced food intake or respiratory distress) = 1, severely sick (reduced food intake and respiratory distress) = 2, dead = 3.

**FIGURE 4:**
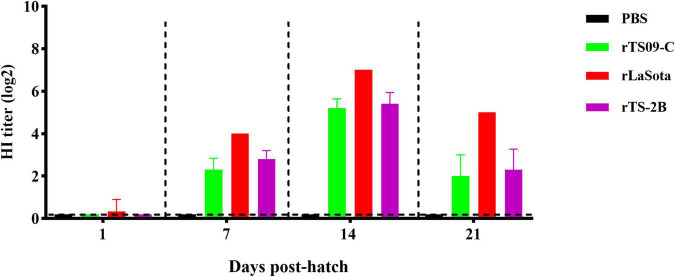
NDV-specific hemagglutinin-inhibition antibody titers in immunized birds. Blood sample from five birds in each group were collected on the day of 1, 7, 14, and 21 dph. The antigen used for HI detection was from the LaSota strain.

To confirm that Fcs is the crucial site regulating the attenuation of TS09-C in chicken embryos, 18-day-old SPF chicken embryos were inoculated with rTS09-C, rLaSota, rTS-2B, or PBS. The survival rates of the rTS-2B group, rLaSota group, and rTS09-C group were 37.5, 4.2, and 91.7%, respectively, at 14 dph (Table 3). The pathogenicity of rTS-2B in chicken embryos differed completely from that of its parental strain TS09-C, but was similar to that of rTS-L-F (survival rate of 23.3%). Although the survival rate of group rTS-2B was greatly higher than that of the rLaSota group, the morbidity in the rTS-2B group was 100%, and all the birds showed disease symptoms (Figure 3). No sick birds were observed after hatching in the rTS09-C group, except for two birds that did not hatch. The HI titer was highest in the rLaSota group, but was similar in the rTS-2B and rTS09-C groups (Figure 4).

**TABLE 3:** Hatchability and survival rates of SPF chickens vaccinated *in ovo* with different NDV mutants.

Vaccines	Dosage (lg EID_50_)	Eggs	%hatched	%survival (14 days post-hatch)
PBS	–	30	96.3(26/27)	91.7(22/24)
rLaSota	3.0	30	33.3(9/27)	4.2(1/24)
rTS09-C	3.0	30	92.6(25/27)	91.7(22/24)
rTS-2B	3.0	30	55.6(15/27)	37.5(9/24)

*Thirty 18-day-old SPF chicken embryos were inoculated with 0.1 mL of PBS, rLaSota, rTS09-C, or rTS-2B at a dose of 10^3^.^0^ EID_50_/egg via the amniotic route. Three birds from each group were killed, respectively, at 2 and 4 dpi, and hatchability was calculated according to the total number of 27 birds. The 14-day survival rate was calculated in 24 birds that were not killed.*

**FIGURE 5:**
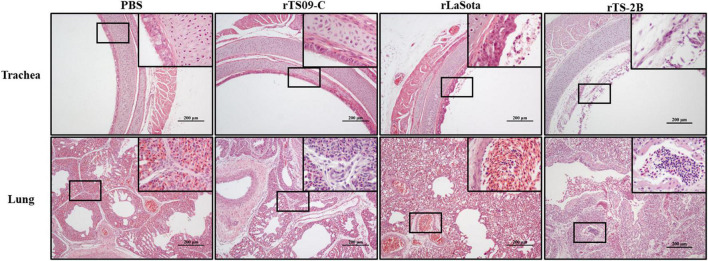
Histopathological analysis of tissue samples from SPF chickens vaccinated *in ovo* with different NDVs. SPF chicken embryos were inoculated with NDVs rLaSota, rTS09-C, or rTS-2B or PBS. Lung and tracheal samples were collected from the *in ovo* vaccinated birds at 4 dpi, fixed in 4% paraformaldehyde, paraffin embedded, sectioned, stained with hematoxylin–eosin, and analyzed microscopically. Scale bar = 200 μm.

The histopathological lesions in the respiratory tissues (lung and trachea) from each group were evaluated at 4 dpi. As shown in Figure 5, no histopathological lesions were observed in both lung and tracheal tissues from the PBS or rTS09-C group. Moderate histopathological lesions were observed in both the lung (hemorrhage, congestion, and lymphocyte infiltration) and tracheal tissues (ciliary shedding, mucosal epithelial cell necrosis) in birds from the rLaSota group. Similarly, the hemorrhage, congestion, and lymphocyte infiltration in lungs, the ciliary shedding and mucosal epithelial cell necrosis in tracheas of birds from the rTS-2B group were observed. These results demonstrated that mutant strain rTS-2B was pathogenic when used as an *in ovo* vaccine for SPF chickens, which is consistent with the results of survival rate. These results further confirm the Fcs is the crucial attenuation determinant of TS09-C in chicken embryos.

### rTS-2B Has Similar Tissue Tropism to rLaSota

**FIGURE 6:**
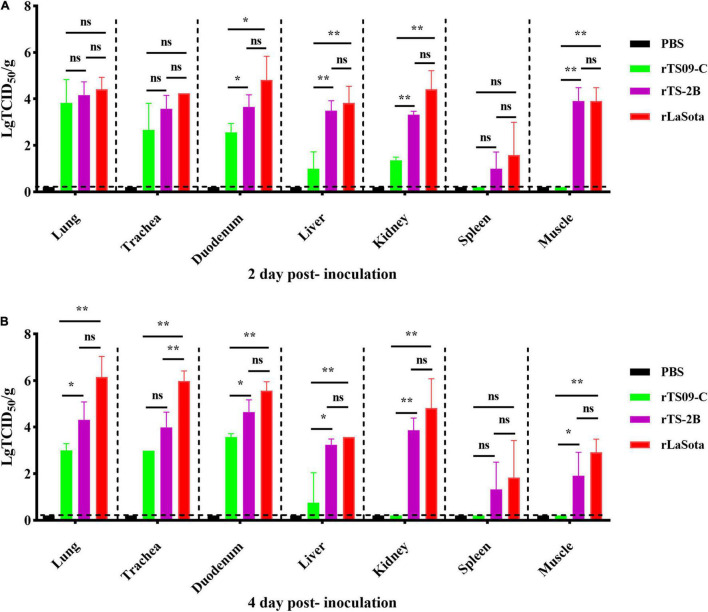
Viral titers in different tissues of chickens inoculated with different NDV strains. SPF chicken embryos were inoculated with NDVs rLaSota, rTS09-C, rTS-2B, or PBS. Lung, trachea, duodenum, liver, kidney, spleen, and muscle samples were collected from birds vaccinated *in ovo*, at 2 **(A)** and 4 dpi **(B)**. The viral titer of each sample was determined in BHK21 cells. Statistical significance in the viral titers was determined with a two-tailed *t* test (ns, *p* > 0.05; *p < 0.05; **p < 0.01).

Tissue tropism of NDV in chicken embryo was determined by viral titration in the lung, trachea, duodenum, liver, kidney, muscle, and spleen samples from the killed birds at 2 and 4 dpi. As shown Figure 6, the distributions the viruses were detected in all collected tissues from the rLaSota and rTS-2B groups at 2 and 4 dpi, and was detected in only partial tissues from the rTS09-C group (lung, trachea, duodenum, liver, and/or kidney). At 2 and 4 dpi, the virus titers of duodenum, liver, kidney and muscle were no statistically significant difference between rTS-2B and rLaSota groups (*p* > 0.05), but the virus titers of these tissues in rTS-2B group were significantly higher than rTS09-C group (*p* < 0.05). In addition, the virus titer of lung in rTS-2B group also showed no statistically significant difference comparing rLaSota group (*p* > 0.05), and significantly higher than rTS09-C group at 4 dpi (*p* < 0.05). These results demonstrated that the tissue tropism of rTS-2B was similar to that of rLaSota and caused systemic infection in chicken embryo.

## Discussion

In this study, we investigated the molecular mechanism underlying the attenuation of NDV in chicken embryo when used as an *in ovo* vaccine. The F gene was shown to be the crucial attenuation determinant of NDV in chicken embryos by evaluating the safety of chimeric NDV strains exchanging the F genes in NDV strains LaSota and TS09-C, as *in ovo* vaccines. The Fcs was confirmed as the key site determining the attenuation of NDV in chicken embryos when point mutations were introduced into it. Finally, we showed that rTS-2B has similar tissue tropism to that of rLaSota, which may be associated with the pathogenicity of NDV in chicken embryos.

The virulence of NDV is predominantly dependent on the F and HN proteins. The HN protein is involved in receptor binding and the release of the virus, and the F protein mediates the fusion of the virion envelope with the host-cell plasma membrane (Morrison, 2003; Samal et al., 2011; Yu et al., 2017; Yan et al., 2020). The F protein is synthesized as an inactive precursor, F0, which is cleaved at Fcs by a cellular protease into the activated forms (or subunits), F1 and F2 (Samal et al., 2011; Ganar et al., 2014). Here, we have shown that the Fcs containing three basic amino acids is the key attenuation determinant of NDV in chicken embryos when NDV was used as an *in ovo* vaccine. The amino acid sequence at Fcs is a key determinant of NDV virulence, modification of the Fcs can significantly alter the fusion and virulence of NDV (Wang et al., 2017a). There are totally 3,903 complete F protein sequences in the GenBank database. Based on the number of basic amino acids at cleavage site (112–117), the 3903 F protein sequences were classified into five types. As shown in Table 4, the occurrence of two, four, or five basic amino acids in the Fcs was common, occurring in 23.32, 62.36, and 10.30% of all the F protein sequences, respectively. However, the Fcs with one or three basic amino acids was present in fewer isolates, occurring in 1.67 and 2.35% of the sequences examined, respectively. NDV strains containing four or five basic amino acids in the Fcs are virulent strains, and strains containing two or three basic amino acids are avirulent strains (Kattenbelt et al., 2006; Kang et al., 2016; Wang et al., 2017b). The virulence phenotype of strains containing only one basic amino acid in the Fcs is unknown.

**TABLE 4:** Summary of sequence information at the NDV F protein cleavage site.

Number of basic amino acids^a^	Sequence number^b^	Percentage	Information of representative strain				
			Name	Sequence in Fcs	Accession number	ICPI	MDT/MLD^c^
1	65	1.67%	ND31	EQQERL	ADC40755	–	–
2	910	23.32%	LaSota	GRQGRL	JF950510	0.4	103
3	92	2.35%	**TS09-C**	**GKQRRL**	**JX110635**	**0.00**	**>168**
4	2434	62.36%	Texas/GB	RRQRRF	GU978777	1.75	55
5	402	10.30%	rNDV-Q114R	RRRRRF	NA^d^	1.33	–

*^a^Number of basic amino acids at F protein sites 112–115. ^b^Total number of sequences was 3,903. ^c^In hours. ^d^Sourced from reference Doi: 10.1099/vir.0.033399-0. TS09-C is the attenuated NDV strain used in this study, and it is also the representative NDV strain containing three basic amino acids in fusion protein cleavage site.*

The pathogenic mechanism of NDV in hatched birds is well-known. Typical virulent strains have the virulent Fcs containing four basic amino acids, whereas the lentogenic strains have the avirulent Fcs, which contains only two basic amino acids (Wang et al., 2017b; Liu et al., 2018). The velogenic NDV strains can be cleaved intracellularly by ubiquitous furin-like proteases, causing a systemic infection in the chickens, lentogenic strains are cleaved extracellularly by trypsin-like proteases in the respiratory and intestinal tracts, and only causes local infections (Dortmans et al., 2011; Liu et al., 2018; Bello et al., 2020). However, the pathogenic mechanism of NDV in chickens before they hatch was poorly understood. We have shown that NDV with Fcs containing two basic amino acids, such as strain rLaSota and rTS-2B, is highly pathogenic in chicken embryos, causing moderate respiratory tissue lesions and system infection. While NDV with Fcs containing three basic amino acids, such as strain rTS09-C, is attenuated in chicken embryos, and only causes local infection, and no histopathological lesions were observed in lungs or tracheas of the inoculated birds. It was speculated that different proteases are involved in the cleavage of F proteins with Fcs containing two and three basic amino acids, and the distributions of these proteases in chicken embryos lead to the differences in the viral tissue tropism and virulence in chicken embryos. Identifying the proteases that cleave the TS09-C F protein is the direction of our future research.

The *in ovo* vaccination technology has evolved from a laboratory concept to a commercially available system within a decade, with application to 90% of United States broiler production a few years later (Williams and Zedek, 2010). The automation is requisite in commercial application. Automated *in ovo* vaccination has the capacity to administer a vaccine anywhere and immunize 25,000–62,000 eggs per hour, reducing both labor costs and the risk of human error (Peebles, 2018). The chicken embryo is immunocompetent and capable of producing both an innate and an adaptive immune response to pathogens during late embryonic development. Therefore, *in ovo* vaccination can induce an earlier immune response (Peebles, 2018; Schilling et al., 2018; Vaezirad et al., 2018; Hincke et al., 2019).

In summary, we previously confirmed that NDV strain TS09-C has potential utility as an *in ovo* vaccine for NDV (Wen et al., 2017; Fan et al., 2020). Here, we confirmed that the Fcs containing three basic amino acids is the critical determinant of the attenuation of NDV in chicken embryos. This finding extends our understanding of the pathogenic mechanism of NDV in chicken embryos, and should promote the development of *in ovo* vaccines for NDV.

## Data Availability Statement

The original contributions presented in the study are included in the article/supplementary material, further inquiries can be directed to the corresponding authors.

## Ethics Statement

The animal study was reviewed and approved by the Institutional Animal Care and Use Committee of the Hubei Academy of Agricultural Sciences, Wuhan, China.

## Author Contributions

GC, GW, and HF designed the experiments. HF, LL, SF, XS, YX, and XR performed the experiments. HF, YS, and ZZ analyzed the data. HF and GW wrote the manuscript. GW directed the project. All authors contributed to the article and approved the submitted version.

## Conflict of Interest

The authors declare that the research was conducted in the absence of any commercial or financial relationships that could be construed as a potential conflict of interest.

## Publisher’s Note

All claims expressed in this article are solely those of the authors and do not necessarily represent those of their affiliated organizations, or those of the publisher, the editors and the reviewers. Any product that may be evaluated in this article, or claim that may be made by its manufacturer, is not guaranteed or endorsed by the publisher.
